# Blood Lead Levels and Associated Sociodemographic and Occupational Factors in Children from Two Mexican Communities: A Comparative Study

**DOI:** 10.3390/toxics14090744

**Published:** 2026-08-23

**Authors:** Jesús García-Gómez, Oscar Humberto Rodríguez-Aguirre, Jorge Adrian Ruíz-Romero, Elvia Harumi Scott-López, Erick Jonathan Becerra-Iñiguez, María Claudia Espinel-Bermúdez, Arnulfo Hernán Nava-Zavala, Adriana Aguilar-Lemarroy, Augusto Sarralde-Delgado, Luis Felipe Jave-Suárez

**Affiliations:** 1 Centro de Investigación Biomédica de Occidente (CIBO), División de Inmunología, Instituto Mexicano del Seguro Social (IMSS), Guadalajara 44340, Jalisco, Mexico; jesus.garcia9891@alumnos.udg.mx; 2 Programa de Doctorado en Ciencias Biomédicas, Centro Universitario de Ciencias de la Salud, Universidad de Guadalajara, Guadalajara 44340, Jalisco, Mexico; 3 Unidad de Medicina Familiar No. 93, Servicio de Medicina Familiar, Instituto Mexicano del Seguro Social (IMSS), Tonalá 45400, Jalisco, Mexico; osukaru01@hotmail.com; 4 Unidad de Medicina Familiar No. 171, Servicio de Medicina Familiar, Instituto Mexicano del Seguro Social (IMSS), Zapopan 45070, Jalisco, Mexico; jorgearuizromero@gmail.com; 5 Laboratorio de Salud en el Trabajo, Instituto Mexicano del Seguro Social (IMSS), Guadalajara 44340, Jalisco, Mexico; elvia.scott@imss.gob.mx (E.H.S.-L.); erick.becerra@imss.gob.mx (E.J.B.-I.); 6 Unidad de Investigación Biomédica 02, Centro Médico Nacional de Occidente, Instituto Mexicano del Seguro Social (IMSS), Guadalajara 44340, Jalisco, Mexico; maria.espinel@imss.gob.mx; 7 Unidad de Investigación Epidemiológica y en Servicios de Salud, Centro Médico Nacional de Occidente, Instituto Mexicano del Seguro Social (IMSS), Guadalajara 44340, Jalisco, Mexico; navazava@yahoo.com.mx (A.H.N.-Z.); adriana.aguilar@academicos.udg.mx (A.A.-L.); 8 Programa Internacional de Medicina, Universidad Autónoma de Guadalajara, Zapopan 45129, Jalisco, Mexico; 9 Departamento de Inmunología y Reumatología del Hospital General de Occidente, Secretaría de Salud Jalisco, Guadalajara 45170, Jalisco, Mexico; 10 Órgano Operativo de Administración Desconcentrada Jalisco, Instituto Mexicano del Seguro Social, Guadalajara 44340, Jalisco, Mexico

**Keywords:** blood lead level, glazed ceramics, pediatric exposure, environmental health surveillance, Mexico

## Abstract

Lead is a neurotoxic metal. No safe blood lead level (BLL) has been identified in children; the CDC reference value was lowered to 3.5 µg/dL in 2021, while Mexico’s regulatory threshold remains at 10 µg/dL. Lead-glazed earthenware is the main documented source of pediatric exposure in Mexico. We studied children 0–15 years insured by the Mexican Social Security Institute (IMSS) in two Jalisco municipalities: Tonalá (*n* = 500), historically a ceramic-producing center, and Zapopan (*n* = 374), an urban area without this tradition. BLL was measured by graphite furnace atomic absorption spectrophotometry, and sociodemographic, environmental, occupational, and household exposure data were collected by questionnaire and analyzed with bivariate tests and multivariable models. Median BLL was 1.16 µg/dL in Tonalá and 2.08 µg/dL in Zapopan. Few children reached 5 µg/dL (3.2% and 6.4%), roughly twice as many exceeded 3.5 µg/dL (6.6% and 16.3%), and most exceeded 1 µg/dL (59.4% and 93.6%). In Zapopan, household glazed earthenware use and younger age were independently associated with a higher BLL in every model; in Tonalá, paternal professional occupation was associated with a lower BLL, an exploratory finding given its small subgroup. Widespread low-level exposure at both sites supports sustained facility-level surveillance and alignment of Mexico’s regulatory criterion with current reference values.

## 1. Introduction

Environmental pollution is now one of the leading drivers of disease and premature death worldwide. Among heavy metals, lead (Pb) stands out for its persistence, toxicity, and tendency to accumulate in the body [1]. Most countries have phased lead out of gasoline and paint, yet chronic exposure to low concentrations remains a significant public health problem, especially in low- and middle-income countries [2].

Lead is a neurotoxicant that can permanently impair children’s cognitive and behavioral development. Children absorb more lead through the gut than adults, and behaviors typical of early childhood, like hand-to-mouth activity and pica, make them more likely to ingest contaminated dust and particles [2]. Decades of research now support what the American Academy of Pediatrics has stated formally: no safe level of childhood lead exposure exists [3]. The CDC responded to this evidence in October 2021, lowering its blood lead reference value from 5 to 3.5 µg/dL [4]. Mexico’s regulatory threshold has not kept pace: NOM-199-SSA1-2000 still sets 10 µg/dL as the maximum level for the non-occupationally exposed population [5].

Mexico’s National Health and Nutrition Survey (ENSANUT 100k) found that 21.8% of children aged 1–4 in localities with fewer than 100,000 residents had blood lead levels ≥ 5 µg/dL [6]. A subsequent national extrapolation put the figure at 17.4% for the same age group, corresponding to roughly 1.4 million children [7]. The main exposure sources identified in Mexico include lead-glazed earthenware used for cooking and food storage, occupational exposure in traditional artisanal workshops, and environmental contamination linked to industrial sites and waste management [6,8].

Within Jalisco, a state in west-central Mexico, the municipality of Tonalá, part of the Guadalajara metropolitan area, illustrates this problem clearly. Tonalá is a traditional artisanal center where families produce lead-glazed earthenware in workshops often attached to their homes. Studies from the 1980s found extremely high blood lead levels in the children of local potters [9]. Other environmental hazards compound this traditional exposure source: landfills, metal and electronic waste recyclers, and the heavily contaminated Río Santiago basin are all concentrated in Tonalá [10,11]. Zapopan, a neighboring municipality within the same metropolitan area, presents a contrasting profile: a commercial and industrial economy with a scarce pottery-making tradition, though households there still commonly use glazed earthenware. Neither municipality’s pediatric lead exposure has been characterized among children covered by the Mexican Social Security Institute (IMSS), the social security institution that covers private-sector formal employees and their families and serves a large share of Mexico’s population.

To address this gap, we studied children at IMSS Family Medicine Units (Unidades de Medicina Familiar, UMF, by their Spanish acronym) No. 93 and No. 171, serving Tonalá and Zapopan, respectively. The two municipalities were selected because they differ in the exposure conditions of interest while sharing a metropolitan location and the same tier of institutional healthcare; Tonalá has produced lead-glazed earthenware for generations, whereas Zapopan has no comparable local production despite widespread domestic use of the same objects. Comparing them tests whether the exposure factors associated with the blood lead level differ according to whether a community produces glazed ceramics or only uses them. Applying the same recruitment procedure, laboratory method, questionnaire, and analytical specification at both sites allows that comparison to rest on the sites themselves rather than on differences in how they were studied. This facility-level approach addresses a gap left by national and regional surveys, which cannot resolve exposure patterns at the level of the communities a clinic actually serves. Accordingly, this study aimed to characterize blood lead levels and their associated factors among IMSS-insured children at UMF No. 93 (Tonalá) and UMF No. 171 (Zapopan).

## 2. Materials and Methods

### 2.1. Study Population

This study was conducted at two IMSS clinics in Jalisco, Mexico. Both sites used identical eligibility criteria: children aged 0–15 years, of either sex, insured by IMSS, with informed consent from a parent or guardian, and assent from children 8 years or older. We excluded children with chronic kidney disease, those unable to provide assent, and any case with an incomplete questionnaire or an insufficient blood sample. Participants were recruited at both sites by consecutive sampling among eligible children already scheduled for a complete blood count as part of routine care, with the lead determination added to the same venipuncture; no family declined the additional measurement. At UMF No. 93 in Tonalá, 500 children were enrolled between November 2024 and August 2025 (calculated target, 419). At UMF No. 171 in Zapopan, 374 children were enrolled between April 2023 and June 2024 (calculated target, 373). Sample sizes were calculated using the formula for estimating a proportion in a finite population, assuming an expected prevalence of 17% [7] and 95% confidence, applied to the pediatric population registered at each unit.

### 2.2. Variables and Measurements

Blood lead level (BLL), expressed in µg/dL, served as the dependent variable in both sites. We quantified it using graphite furnace atomic absorption spectrophotometry (iCE 3500 AA System, Thermo Fisher Scientific, Waltham, MA, USA; λ = 283.3 nm), drawing 3 mL of venous blood into EDTA tubes from each participant. The method was implemented following Appendix A of NOM-199-SSA1-2000 [5]; the limit of detection (LOD) and limit of quantification (LOQ) were established according to the Eurachem guide on method validation [12] and corresponded to 0.25 and 0.82 µg/dL, respectively. Values below the LOQ were retained as reported by the laboratory; 26.8% of samples at UMF No. 93 and 2.9% at UMF No. 171 fell below the LOQ, and one sample per site fell below the LOD (0.22 and 0.24 µg/dL). Readings remain available below these limits, though with increasing relative uncertainty; consistent with Eurachem guidance, LOD and LOQ define the concentrations at which detection and quantification meet defined performance criteria, rather than a floor below which no measurement is obtained. Blood lead determinations were performed in the IMSS Occupational Health Laboratory under a quality assurance protocol aligned with NOM-199-SSA1-2000 [5], specifying calibration curves with a correlation coefficient of at least 0.9980, prepared from a lead standard traceable under ISO 17034:2016 [13] and ISO/IEC 17025:2017 [14]; a calibration blank every five samples, with an acceptance limit of 0.0015 absorbance units; a certified whole-blood lead control every 20 samples, with recalibration and batch reanalysis if the control result fell outside its certified interval; duplicate analyses with a maximum permissible difference of 10% and a maximum instrumental coefficient of variation of 10%; and a certified reference material at the start of each run (lyophilized human blood, nominal lead concentration 52 µg/L; Joint Research Centre, European Commission, ID 636) with an acceptance criterion of ±10% of the nominal value.

For descriptive purposes, we classified BLL into five risk categories (<1, 1–3.5, 3.5–5, 5–10, and ≥10 µg/dL, with lower bounds inclusive and upper bounds exclusive) and reported prevalence at four thresholds: ≥1 µg/dL, ≥3.5 µg/dL (the current CDC blood lead reference value [4]), ≥5 µg/dL (retained as a benchmark for comparison with ENSANUT and earlier literature [15]), and ≥10 µg/dL (the action level under NOM-199-SSA1-2000 [5]).

Parental questionnaires captured four categories of predictor variables. Environmental exposure was defined as the respondent reporting a probable source of contamination within 500 m of the home, such as a recycling center, pottery workshop, metal-processing facility, or landfill. Occupational exposure was defined as either parent working in an activity with recognized lead exposure, such as pottery-making, recycling, battery-handling, painting, or mining. Household exposure captured the use of lead-glazed earthenware for cooking or food storage. We also recorded the child’s age and sex as well as the parents’ education levels. Parental education was analyzed as the highest level reported for either parent. For descriptive stratification, age was additionally grouped using the pediatric age classification proposed by the StaR Child Health Group [16]: infancy, toddler, early childhood, middle childhood, and adolescence. Because age was recorded in completed years, we adapted the original continuous age boundaries to whole-year bins, assigning each child to a single category based on age in completed years at the time of blood sampling.

### 2.3. Statistical Analysis

All analyses were performed in Python 3.13.3, using pandas 2.2.3 and NumPy 2.3.4 for data handling, SciPy 1.16.0 and statsmodels 0.14.6 for statistical modeling, scikit-posthocs 0.11.4 for post-hoc testing, scikit-learn 1.6.1 for classification metrics, and Matplotlib 3.10.3 with Seaborn 0.13.2 for visualization. We described continuous variables using medians and interquartile ranges, given the non-normal distribution of blood lead levels. Proportions are reported with 95% Wilson score confidence intervals. Between-site comparisons used the Mann–Whitney U test for continuous variables and the Pearson chi-square test for proportions, with Fisher’s exact test when any expected cell count fell below five. For within-site comparisons, we used the Mann–Whitney U test for two-group comparisons and the Kruskal–Wallis test with Dunn’s post-hoc and Bonferroni correction for comparisons involving three or more groups. We assessed the relationship between age and blood lead level using Spearman’s correlation coefficient (ρ).

Multivariable analysis applied a single predictor specification at both sites: age, sex, glazed earthenware use, environmental exposure, occupational exposure, paternal occupation, and parental education. Categorical predictors retained all response categories observed at each site as model levels; terms with no variation at a given site were necessarily omitted. Three complementary models were fitted at each site. Multiple linear regression treated blood lead level as continuous, preserving the µg/dL scale. Linear regression on the natural logarithm of blood lead level addressed the right-skewed distribution, with coefficients reported as percent differences in geometric mean; for these models we examined residual, leverage, and Cook’s distance diagnostics. Logistic regression treated blood lead level as binary at site-specific thresholds (≥1 µg/dL at UMF No. 93; ≥2.5 µg/dL at UMF No. 171), selected from each site’s observed distribution because the ≥5 µg/dL threshold yielded too few cases for stable estimation; these models are reported as exploratory, and their odds ratios are not comparable between sites. We considered *p* < 0.05 statistically significant throughout.

### 2.4. Ethical Considerations

Both protocols were classified as minimal risk and approved by the IMSS Local Health Research Committee (Comité Local de Investigación en Salud) No. 1306, Jalisco State Delegation, under registration numbers R-2024-1306-109 (UMF No. 93, Tonalá) and R-2023-1306-083 (UMF No. 171, Zapopan). Both studies followed the principles of the Declaration of Helsinki, and we obtained informed consent from parents or guardians and assent from children as applicable. Results were delivered to the clinic administration at each site for entry into the participant’s clinical record, and parents were informed at enrollment that they should attend a consultation to receive them. Children with elevated concentrations were referred for clinical evaluation and management under the institutional protocol for lead poisoning in children under 15 years, pregnant women, and women who are breastfeeding, which stratifies management by blood lead concentration, from exposure-source identification and environmental counselling at lower concentrations to chelation therapy at the highest.

## 3. Results

### 3.1. Population Characteristics and Between-Site Comparisons

**Figure 1:**
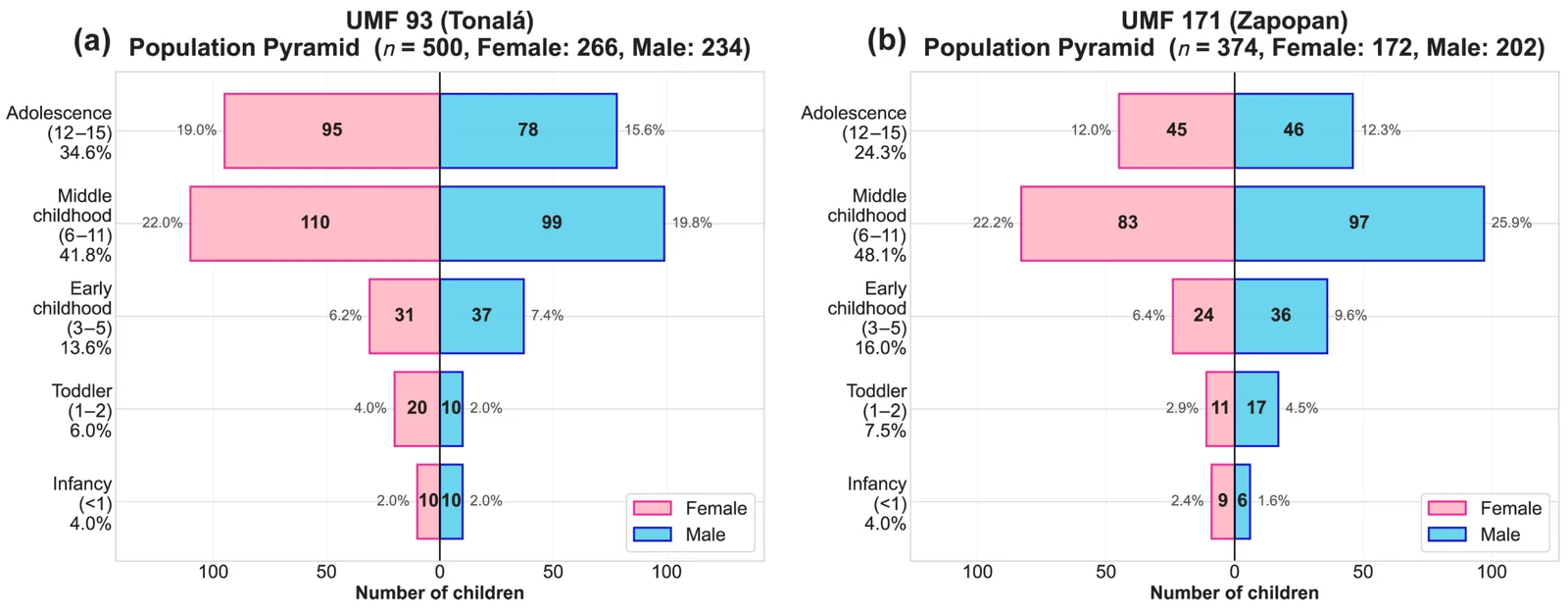
Population pyramid of participants by sex and age group at the two study sites. (**a**) UMF No. 93, Tonalá (*n* = 500). (**b**) UMF No. 171, Zapopan (*n* = 374). Age groups were defined as: infancy (<1 year), toddler (1–2 years), early childhood (3–5 years), middle childhood (6–11 years), and adolescence (12–15 years, truncated at its upper bound per the study’s inclusion criteria). Pink bars represent female participants, and blue bars represent male participants; numbers within each bar indicate absolute frequency, and percentages reflect the proportion of the site total.

We included 874 children aged 0–15 years across the two sites, classified by age using the StaR Child Health pediatric classification. At UMF No. 93 (Tonalá), 500 children were enrolled, 266 girls (53.2%) and 234 boys (46.8%); middle childhood (6–11 years) was the largest age group (209, 41.8%), followed by adolescence (12–15 years; 173, 34.6%) and early childhood (3–5 years; 68, 13.6%), with toddlers and infants together accounting for 10.0%. At UMF No. 171 (Zapopan), 374 children were enrolled, 202 boys (54.0%) and 172 girls (46.0%); middle childhood was also the largest group (180, 48.1%), followed by adolescence (91, 24.3%) and early childhood (60, 16.0%), with toddlers and infants together accounting for 11.5% (Figure 1). Because the study’s upper age limit was 15 years, the adolescence category, which the StaR Child Health classification extends to 18 years, was truncated at its upper bound in both cohorts.

Table 1 summarizes both cohorts side by side, reporting composition, blood lead distribution, exposure indicators, and parental socioeconomic characteristics, with formal between-site comparisons and, for reference, the within-site differences in blood lead level detailed in Section 3.3. The two populations differed modestly in composition: boys were 7.2 percentage points more frequent in Zapopan (*p* = 0.0349), and children in Tonalá were older (median 10 vs. 8 years, *p* = 0.0005). Blood lead levels were substantially higher in Zapopan, with geometric means of 1.22 µg/dL at UMF No. 93 and 2.14 µg/dL at UMF No. 171 (*p* < 0.0001). The difference held across the lower thresholds but not the highest. Levels ≥1 µg/dL were 34.2 percentage points less frequent in Tonalá (59.4% vs. 93.6%, *p* < 0.0001), levels ≥3.5 µg/dL were 9.7 percentage points less frequent (6.6% vs. 16.3%, *p* < 0.0001), and levels ≥5 µg/dL were 3.2 percentage points less frequent (3.2% vs. 6.4%, *p* = 0.0243). The proportion reaching 10 µg/dL did not differ (0.6% vs. 0.8%, *p* = 0.7051).

Exposure profiles contrasted sharply. Environmental exposure was reported 42.1 percentage points more often in Tonalá (72.8% vs. 30.7%, *p* < 0.0001). Glazed earthenware use was 5.9 percentage points less frequent in Tonalá (16.8% vs. 22.7%, *p* = 0.0281), while parental occupational exposure was equally common at both sites (20.2% vs. 20.6%, *p* = 0.8879). Cumulative exposure burden was distributed differently: 14.4% of Tonalá participants reported no exposures compared with 45.2% in Zapopan, while 63.2% reported a single exposure compared with 36.6% (both *p* < 0.0001); the proportion reporting two or more did not differ (22.4% vs. 18.2%, *p* = 0.1271). Parental education and occupation also differed, with higher education (15.4% vs. 28.9%, *p* < 0.0001) and professional paternal occupation (1.8% vs. 4.5%, *p* = 0.0181) both less frequent in Tonalá. Maternal occupation differed in distribution between sites, but blood lead level did not differ across its categories at either site. (*p* = 0.7697 and *p* = 0.5056).

Categorical variables are presented as *n* (% [95% CI]), with confidence intervals calculated using the Wilson score method; continuous variables (age and blood lead level) are presented as medians [IQR]. Blood lead level columns show the median [IQR] within each category. Between sites, continuous variables were compared using the Mann–Whitney U test and proportions using the Pearson chi-square test, with Fisher’s exact test where any expected cell count was below five; a single test is reported for binary variables (sex, environmental exposure, occupational exposure, glazed earthenware use, and each prevalence threshold) and each category was compared separately for variables with three or more categories (cumulative exposure burden, parental education, and parental occupation). Age was compared on the continuous scale rather than by age group, and blood lead risk categories are presented for description only, with the corresponding comparisons reported in the prevalence-at-threshold rows. Comparisons of parental education and parental occupation were restricted to the categories common to both questionnaires, since the “Unknown” category at UMF No. 93, recorded when respondents did not know the parent’s education or occupation, and the “Absent” category at UMF No. 171, recorded when the parent was absent from the household, correspond to distinct responses. Within each site, differences in blood lead level across categories were assessed using the Mann–Whitney U test for two-category variables (sex, environmental exposure, occupational exposure, and glazed earthenware use) and the Kruskal–Wallis test with Dunn’s post-hoc test and Bonferroni correction for three or more categories (age group, cumulative exposure burden, parental education, and parental occupation); superscript letters denote post-hoc groupings, and categories sharing a letter do not differ significantly. Em dashes indicate comparisons not performed. IQR, interquartile range; CI, confidence interval.

### 3.2. Distribution of Blood Lead Levels

**Figure 2:**
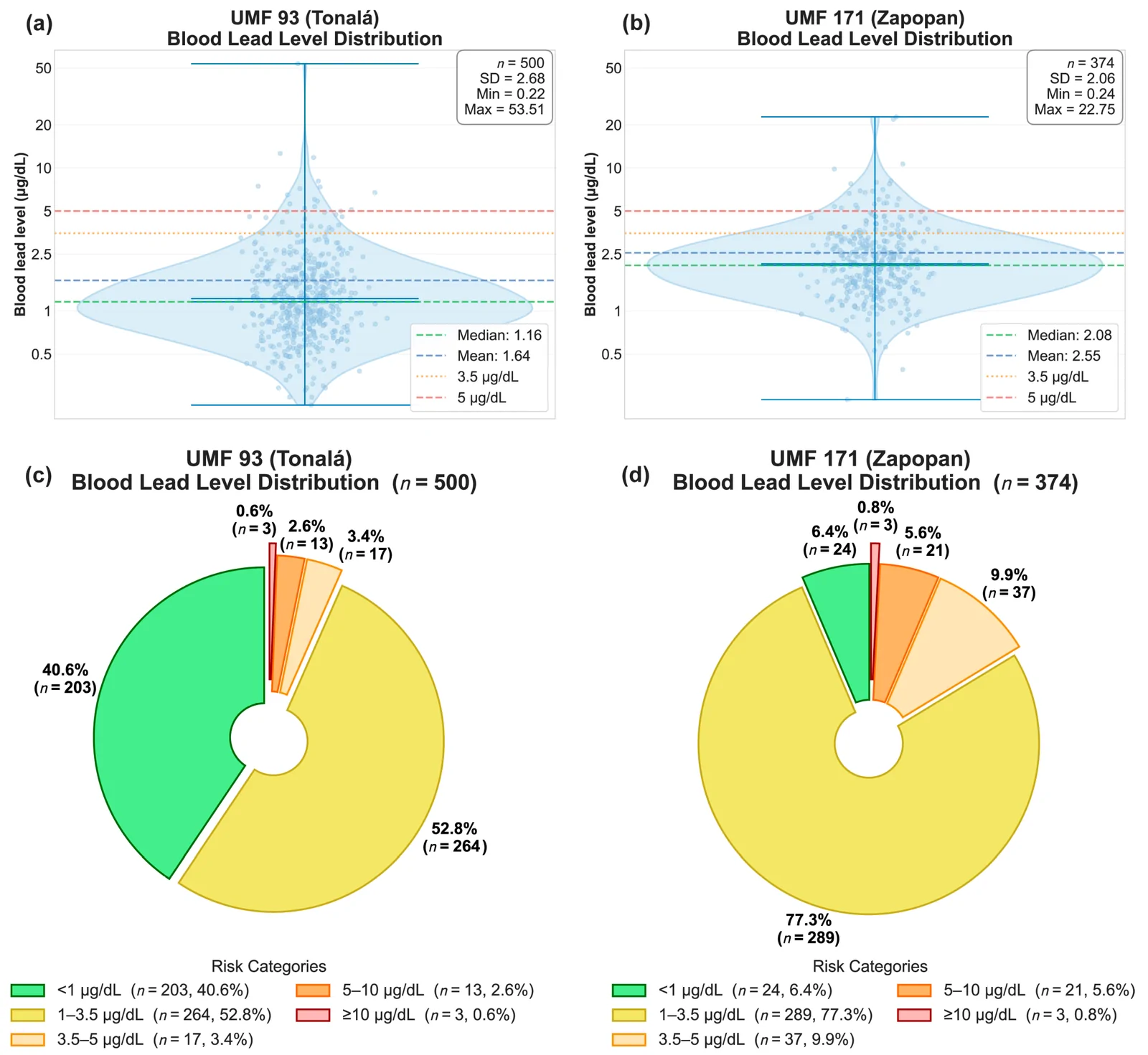
Distribution of blood lead levels at the two study sites. (**a**,**b**) Violin plots showing the density distribution of blood lead levels, presented on a logarithmic y-axis, for UMF No. 93, Tonalá (*n* = 500) and UMF No. 171, Zapopan (*n* = 374), respectively. The dashed green line indicates the median, the dashed blue line indicates the arithmetic mean, the dotted orange line the 3.5 µg/dL reference value, and the dashed red line the 5 µg/dL level. Sample size (*n*), standard deviation (SD), and minimum and maximum values are shown in the upper right of each panel. (**c**,**d**) Distribution of participants by blood lead risk category at the same two sites. Categories were defined as <1, 1–3.5, 3.5–5, 5–10, and ≥10 µg/dL; each segment shows the absolute frequency and percentage of the site total.

Blood lead levels were strongly right-skewed at both sites, and we present them on a logarithmic scale (Figure 2a,b). At UMF No. 93 (Tonalá), the median was 1.16 µg/dL [IQR 0.80–1.77] and the arithmetic mean 1.64 µg/dL (SD 2.68), with values ranging from 0.22 to 53.51 µg/dL. At UMF No. 171 (Zapopan), the median was 2.08 µg/dL [IQR 1.48–3.00] and the arithmetic mean 2.55 µg/dL (SD 2.06), with values ranging from 0.24 to 22.75 µg/dL. Both distributions were centered below 3.5 µg/dL, and in both the mean exceeded the median, reflecting the influence of a small number of high values.

The two cohorts occupied different regions of the risk category distribution (Figure 2c,d). At UMF No. 93, values clustered at the lower end, with 203 children (40.6%) below 1 µg/dL and 264 (52.8%) between 1 and 3.5 µg/dL; the upper categories were sparsely populated, comprising 17 children (3.4%) between 3.5 and 5 µg/dL, 13 (2.6%) between 5 and 10 µg/dL, and 3 (0.6%) at or above 10 µg/dL. At UMF No. 171, the distribution was displaced upward and more compact, with only 24 children (6.4%) below 1 µg/dL and 289 (77.3%) between 1 and 3.5 µg/dL; the upper categories were correspondingly larger, comprising 37 children (9.9%) between 3.5 and 5 µg/dL, 21 (5.6%) between 5 and 10 µg/dL, and 3 (0.8%) at or above 10 µg/dL.

The participant with the highest concentration at UMF No. 93 (53.51 µg/dL) was referred for clinical evaluation under the institutional protocol, with subsequent follow-up. Identified exposure sources included residence in an industrial area and habitual use of glazed earthenware for preparing and displaying food in a family food-vending activity.

### 3.3. Bivariate Analysis of Associated Factors

Having established the overall distributions, we examined how blood lead level varied with sociodemographic characteristics and exposure indicators within each site. Findings are presented by variable, beginning with characteristics of the child and moving toward exposure-specific factors.

**Figure 3:**
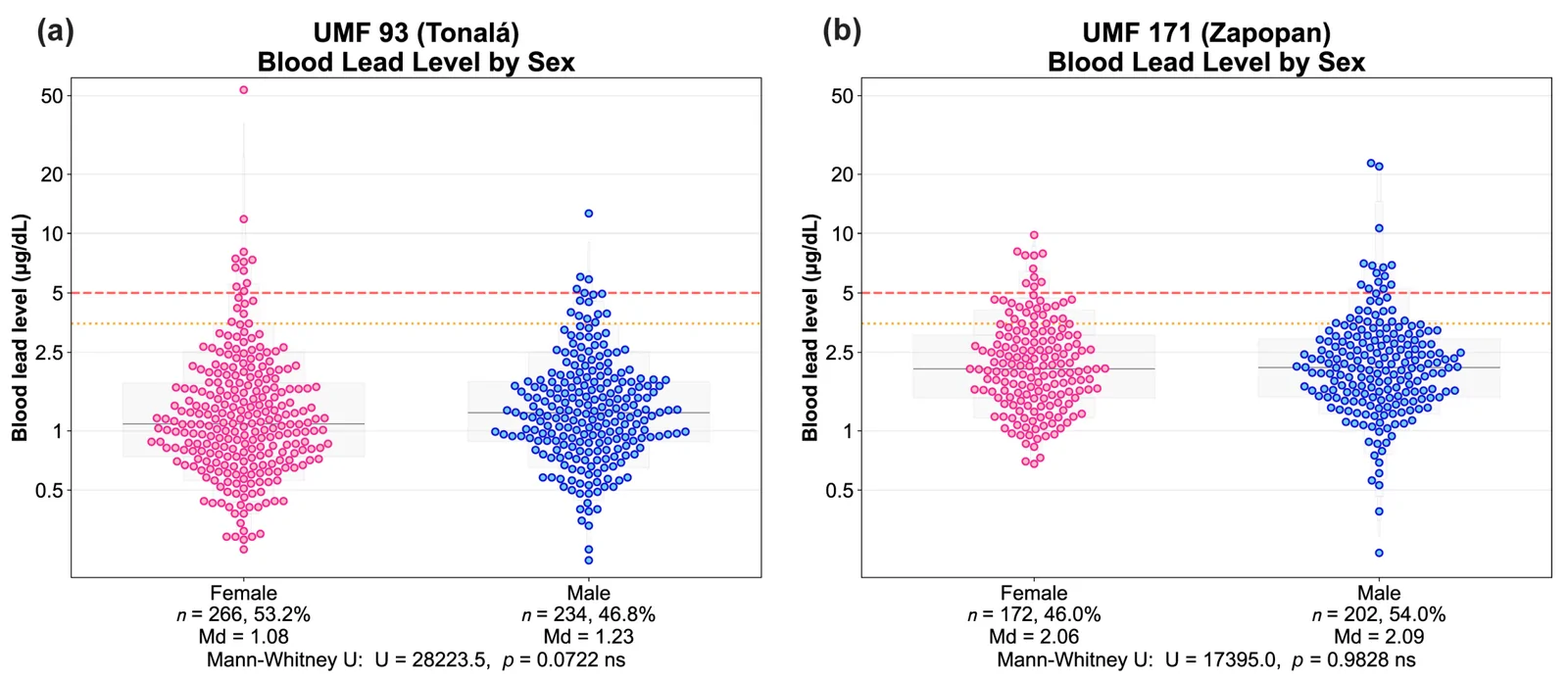
Comparison of blood lead levels by sex at the two study sites. (**a**) UMF No. 93, Tonalá. (**b**) UMF No. 171, Zapopan. Plots show individual participant values (points; pink for female and blue for male) overlaid on a boxplot, with the y-axis on a logarithmic scale. Dotted orange and dashed red lines indicate 3.5 and 5 µg/dL, respectively. Below each category, sample size (*n*), percentage of the site total, and median (Md) are shown. Group comparisons used the Mann–Whitney U test; ns = not significant (*p* > 0.05).

Blood lead levels did not differ significantly between girls and boys at either site (Figure 3). At UMF No. 93, medians were 1.08 µg/dL in girls and 1.23 µg/dL in boys (*p* = 0.0722); at UMF No. 171, medians were 2.06 and 2.09 µg/dL, respectively (*p* = 0.9828).

**Figure 4:**
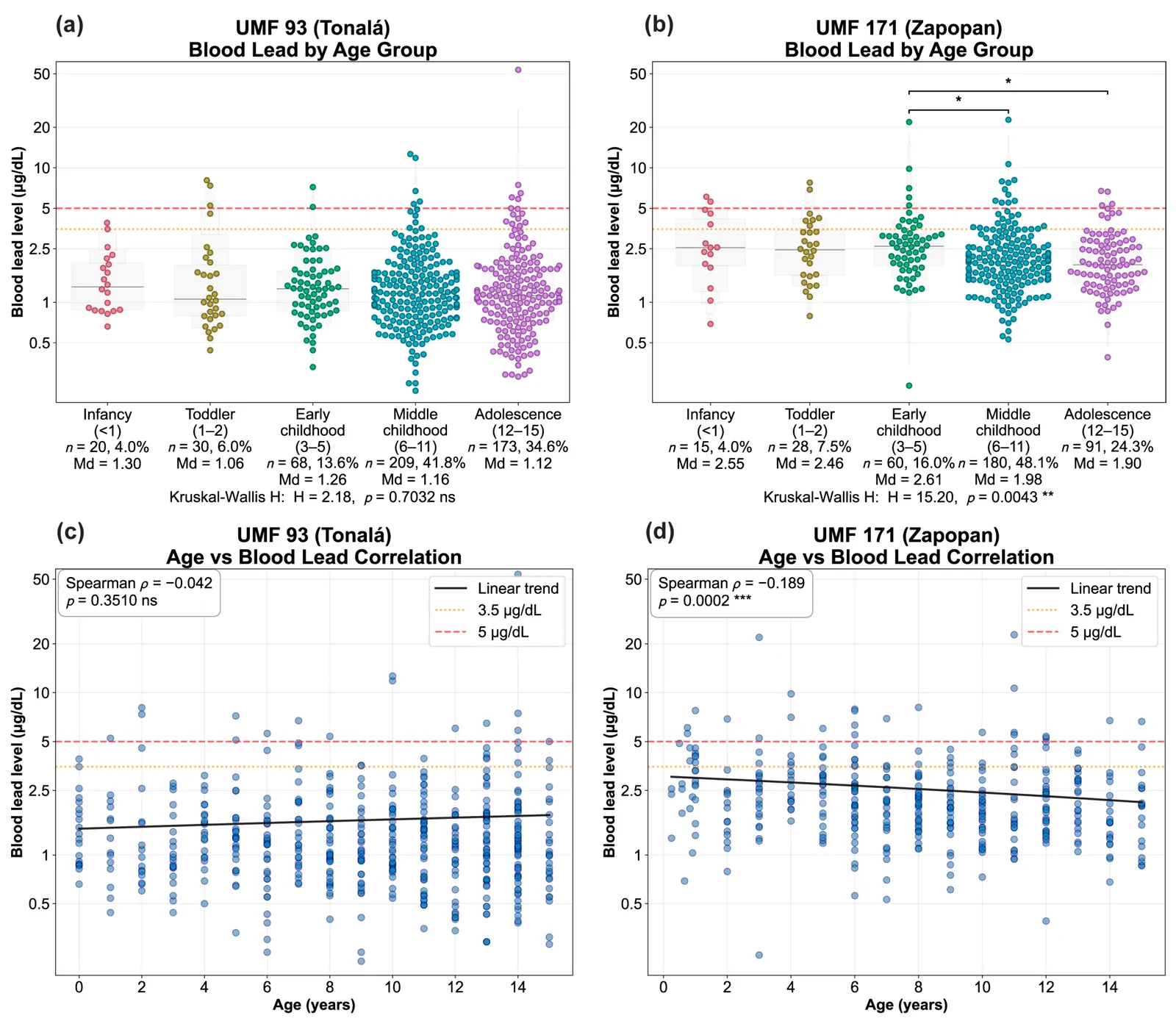
Blood lead level by age group and its correlation with age at the two study sites. (**a**,**b**) Blood lead level by age group for UMF No. 93, Tonalá and UMF No. 171, Zapopan, respectively; age groups follow the classification described in Section 2.2. Below each category, sample size (*n*), percentage of the site total, and median (Md) are shown. Overall group comparisons used the Kruskal–Wallis test; pairwise comparisons used Dunn’s post-hoc test, * *p* < 0.05, ** *p* < 0.01, ns = not significant. (**c**,**d**) Spearman correlation between age and blood lead level for the same two sites, respectively; the y-axis is shown on a logarithmic scale. The solid black line shows the fitted linear trend. Dotted orange and dashed red lines indicate 3.5 and 5 µg/dL, respectively. Spearman’s correlation coefficient (ρ) and its *p*-value are reported; *** *p* < 0.001, ns = not significant.

Stratifying by age group revealed different patterns between the two sites (Figure 4a,b). At UMF No. 93, blood lead levels did not differ significantly across the five age groups (*p* = 0.7032), with medians remaining similar throughout (1.06 to 1.30 µg/dL). At UMF No. 171, the blood lead level differed significantly across age groups (*p* = 0.0043), following a general downward trend with increasing age; early childhood showed the highest median (2.61 µg/dL), while adolescence showed the lowest (1.90 µg/dL). Dunn’s post-hoc comparisons confirmed significant differences between early childhood and middle childhood (*p* < 0.05) and between early childhood and adolescence (*p* < 0.05).

Spearman correlation analysis between age and blood lead level supported these findings (Figure 4c,d). At UMF No. 93, age and blood lead level were not correlated (Spearman ρ = −0.042; *p* = 0.3510). At UMF No. 171, the correlation was statistically significant but weak (ρ = −0.189; *p* = 0.0002), consistent with the downward trend across age groups, though age alone explains only a small proportion of the variability.

**Figure 5:**
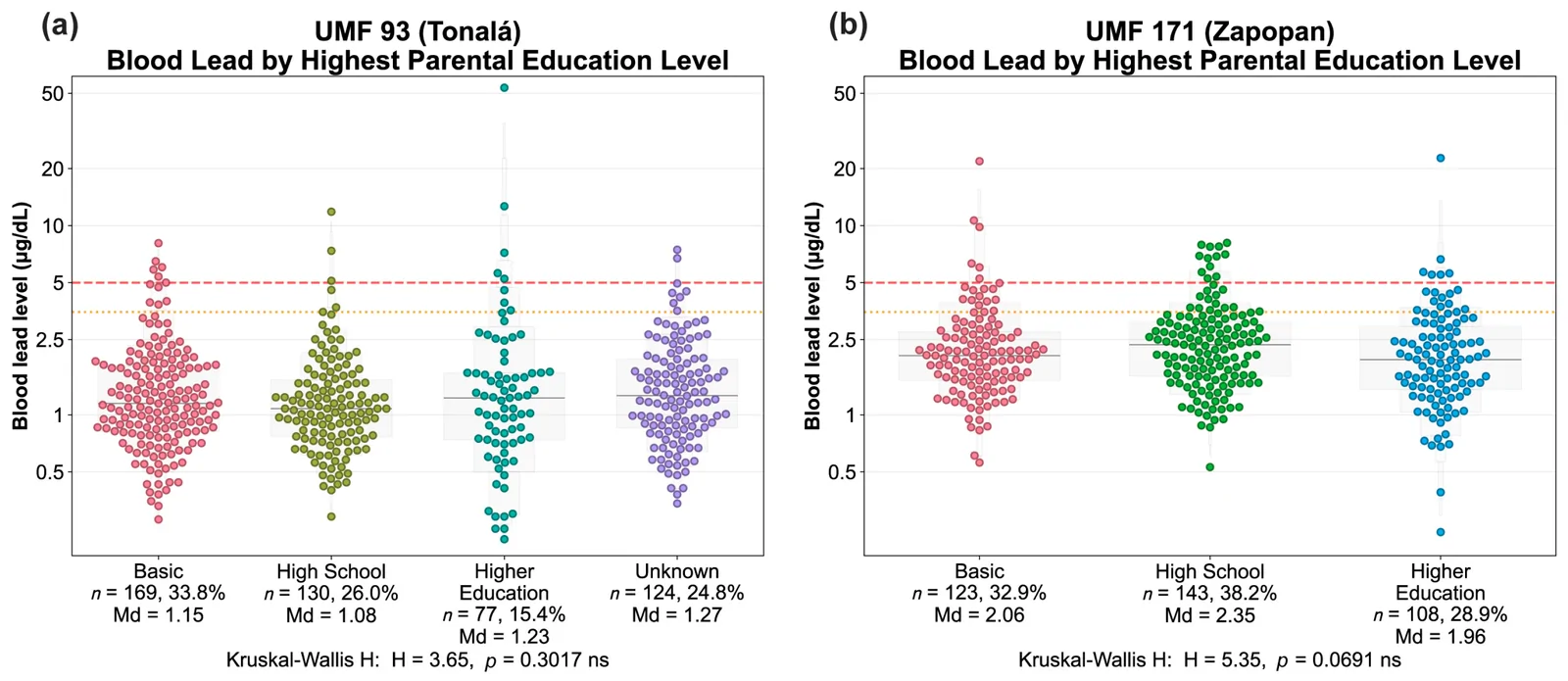
Blood lead level by highest parental education level at the two study sites. (**a**) UMF No. 93, Tonalá. (**b**) UMF No. 171, Zapopan. Plots show individual participant values overlaid on a boxplot, with the y-axis on a logarithmic scale. Dotted orange and dashed red lines indicate 3.5 and 5 µg/dL, respectively. Below each category, sample size (*n*), percentage of the site total, and median (Md) are shown. Group comparisons used the Kruskal–Wallis test; ns = not significant.

Turning to family-environment, we first examined parental education level (Figure 5). On the questionnaire, respondents could report the household’s highest parental education level as basic, high school, higher education, or could fail to specify it; this last option, recorded as “Unknown,” was itself a selectable response and its frequency may carry its own social significance, particularly in relation to the occupation findings below. Parental education level did not differ with blood lead level at either site, though the result at UMF No. 171 approached significance (UMF No. 93: *p* = 0.3017, UMF No. 171: *p* = 0.0691).

**Figure 6:**
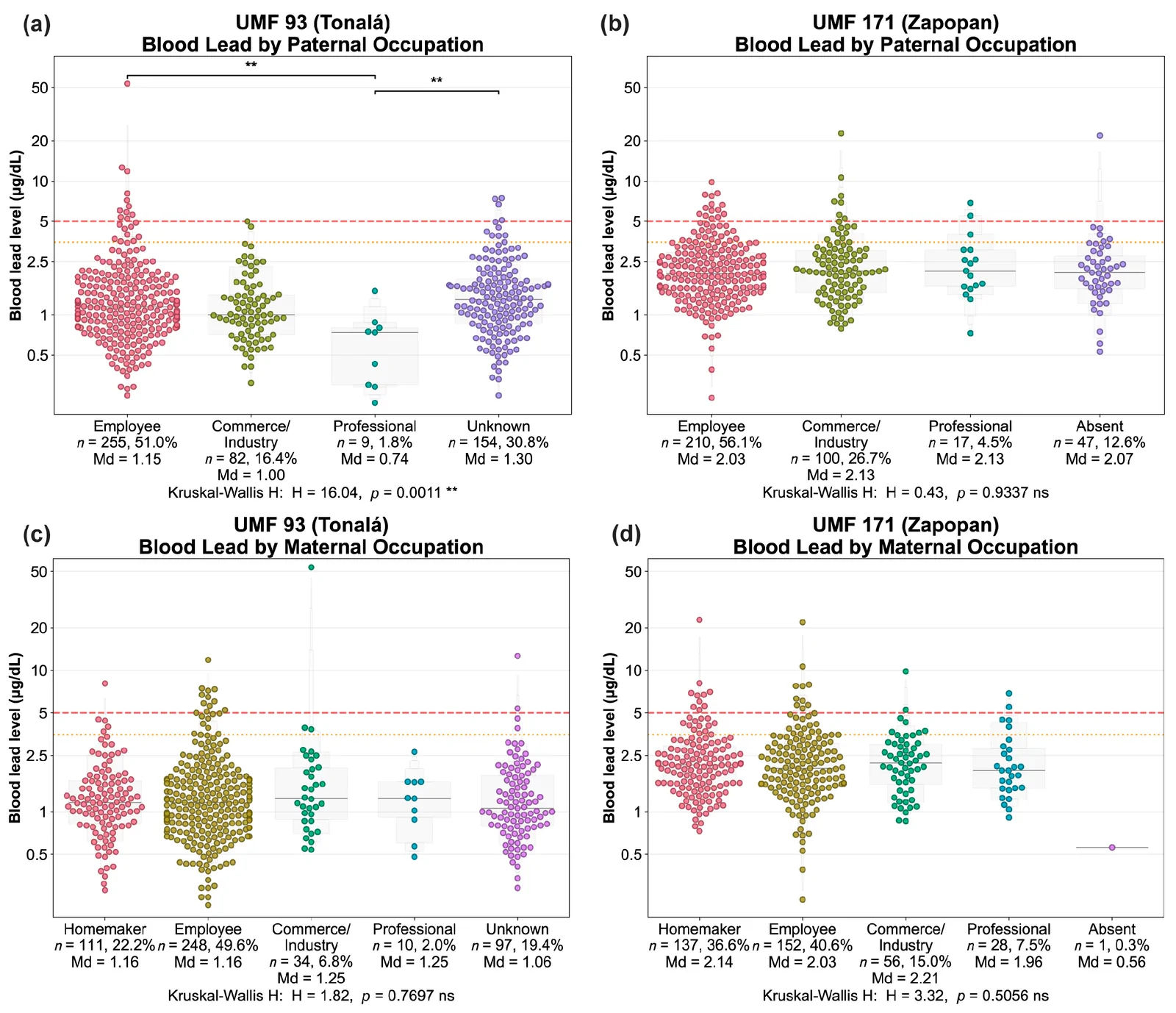
Blood lead level by parental occupation at the two study sites. (**a**,**b**) Paternal occupation for UMF No. 93, Tonalá and UMF No. 171, Zapopan, respectively. (**c**,**d**) Maternal occupation for the same two sites, respectively. Plots show individual participant values overlaid on a boxplot, with the y-axis on a logarithmic scale. Dotted orange and dashed red lines indicate 3.5 and 5 µg/dL, respectively. Below each category, sample size (*n*), percentage of the site total, and median (Md) are shown. Overall group comparisons used the Kruskal–Wallis test; pairwise comparisons used Dunn’s post-hoc test. ** *p* < 0.01, ns = not significant.

Parental occupation is another marker of socioeconomic status and a potential indicator of exposure sources. Paternal occupation differed significantly in association with blood lead level at UMF No. 93, but not at UMF No. 171 (Figure 6a,b). At UMF No. 93 (Kruskal–Wallis *p* = 0.0011), children of fathers in professional occupations had the lowest median (0.74 µg/dL, *n* = 9), differing significantly from employees (*p* = 0.0099), and the “Unknown” category (*p* = 0.0025) but not from commerce/industry. The “Unknown” category comprised the largest share of participants (*n* = 154, 30.8%; recorded when respondents did not know the father’s occupation) and showed the highest median of all categories (1.30 µg/dL). At UMF No. 171, medians were comparable across all categories, including “Absent” (*n* = 47, 12.6%; recorded when the father was absent from the household).

Blood lead level did not differ across maternal occupation categories at either site (UMF No. 93: Kruskal–Wallis *p* = 0.7697; UMF No. 171: Kruskal–Wallis *p* = 0.5056) (Figure 6c,d).

**Figure 7:**
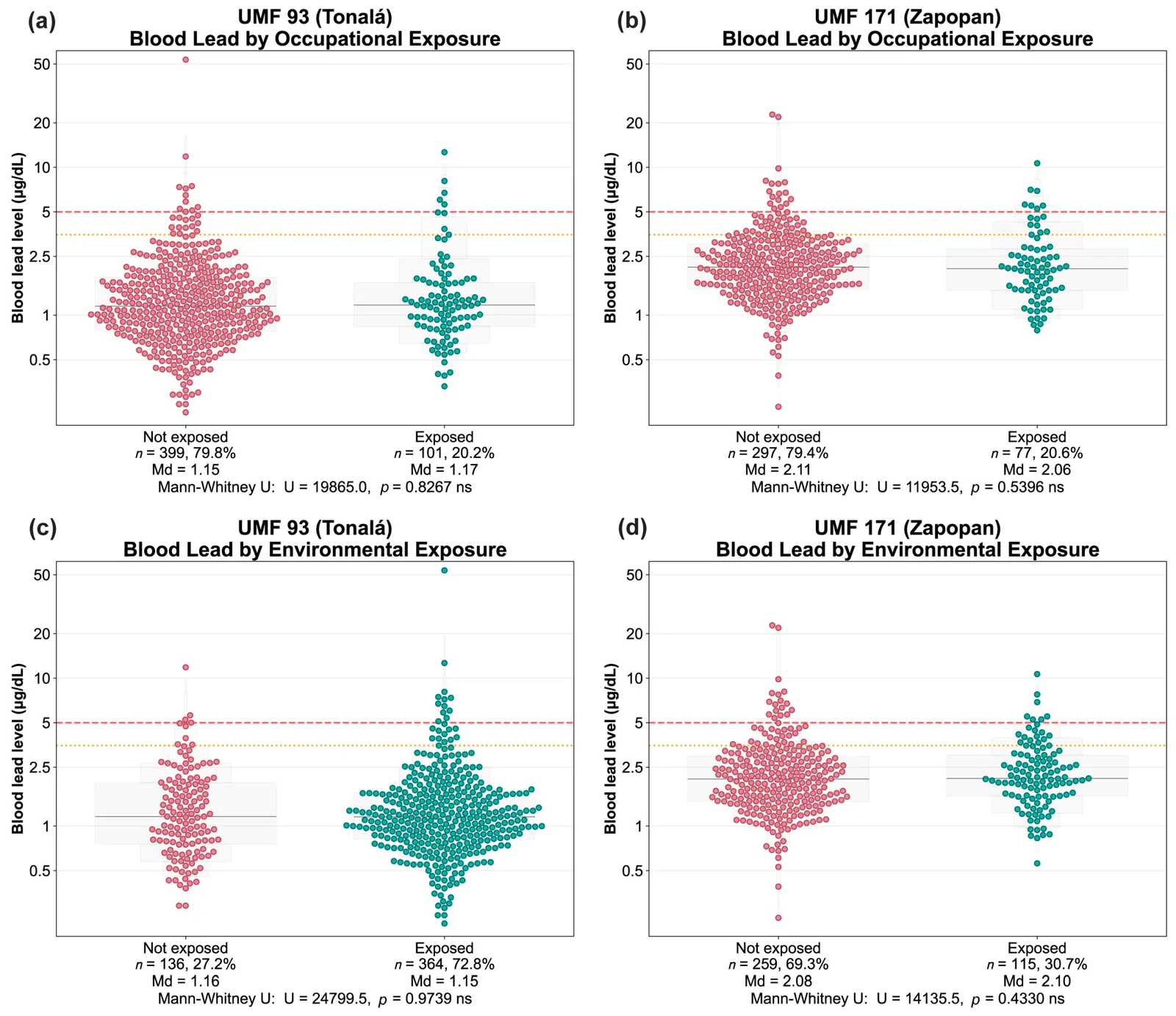
Blood lead level by parental occupational exposure and environmental exposure at the two study sites. (**a**,**b**) Self-reported parental occupational exposure for UMF No. 93, Tonalá and UMF No. 171, Zapopan, respectively. (**c**,**d**) Self-identified environmental exposure (residence within 500 m of a potential lead source) for the same two sites, respectively. Plots show individual participant values overlaid on a boxplot, with the y-axis on a logarithmic scale. Dotted orange and dashed red lines indicate 3.5 and 5 µg/dL, respectively. Below each category, sample size (*n*), percentage of the site total, and median (Md) are shown. Group comparisons used the Mann–Whitney U test; ns = not significant.

Having examined the child’s own characteristics and the broader family environment, we turned to exposure variables specifically linked to lead sources. The first was occupational exposure, defined as either parent’s self-reported occupational exposure to lead (Figure 7a,b). Blood lead level did not differ significantly between children with and without parental occupational exposure at either site (*p* = 0.8267 and *p* = 0.5396, respectively), with medians of 1.15 and 1.17 µg/dL at UMF No. 93 and 2.11 and 2.06 µg/dL at UMF No. 171.

Environmental exposure, defined as a reported lead source within 500 m of the home, likewise did not differ significantly with blood lead level at either site (Figure 7c,d). Medians were 1.16 µg/dL among unexposed and 1.15 µg/dL among exposed children at UMF No. 93 (*p* = 0.9739) and 2.08 and 2.10 µg/dL at UMF No. 171 (*p* = 0.4330).

**Figure 8:**
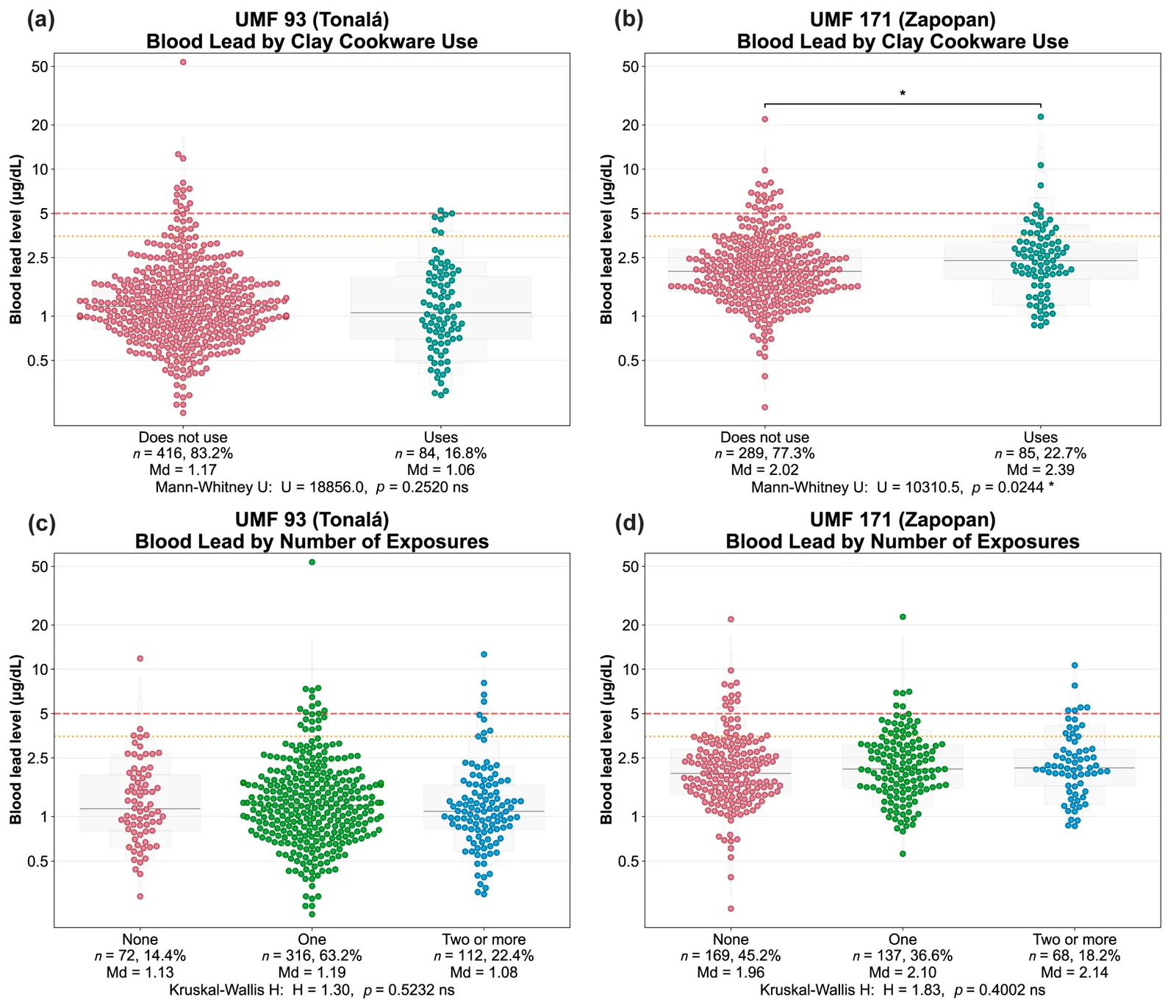
Blood lead level by household glazed earthenware use and cumulative number of reported exposures at the two study sites. (**a**,**b**) Glazed earthenware use for UMF No. 93, Tonalá and UMF No. 171, Zapopan, respectively. (**c**,**d**) Cumulative number of reported exposures (none, one, two or more) for the same two sites, respectively. Plots show individual participant values overlaid on a boxplot, with the y-axis on a logarithmic scale. Dotted orange and dashed red lines indicate 3.5 and 5 µg/dL, respectively. Below each category, sample size (*n*), percentage of the site total, and median (Md) are shown. Group comparisons used the Mann–Whitney U test (**a**,**b**) and Kruskal–Wallis test (**c**,**d**); * *p* < 0.05, ns = not significant.

The third exposure variable was self-reported household use of glazed earthenware (Figure 8a,b). At UMF No. 93, blood lead level did not differ significantly between households that used it and those that did not (1.06 vs. 1.17 µg/dL, *p* = 0.2520). At UMF No. 171, children from households using glazed earthenware had a higher median blood lead level than those from households that did not (2.39 vs. 2.02 µg/dL, *p* = 0.0244).

Finally, blood lead level did not differ across the cumulative number of reported exposures at either site (UMF No. 93: *p* = 0.5232; UMF No. 171: *p* = 0.4002) (Figure 8c,d), with medians ranging from 1.08 to 1.19 µg/dL at UMF No. 93 and from 1.96 to 2.14 µg/dL at UMF No. 171.

### 3.4. Multivariable Modeling of Blood Lead Level

The bivariate analyses above evaluated each factor in isolation. To estimate each variable’s effect while controlling the others, we fit three complementary models at each site using a single predictor specification, described in Section 2.3. Multiple linear regression estimates the average change in blood lead level in µg/dL, preserving the original measurement scale. Logistic regression estimates the odds of exceeding a given concentration, addressing whether a factor distinguishes children above and below a threshold rather than shifting the distribution as a whole. Linear regression on the natural logarithm of blood lead level accommodates the right-skewed distribution and expresses the same effects as percent differences in geometric mean, trading the direct µg/dL interpretation for reduced sensitivity to extreme values.

**Figure 9:**
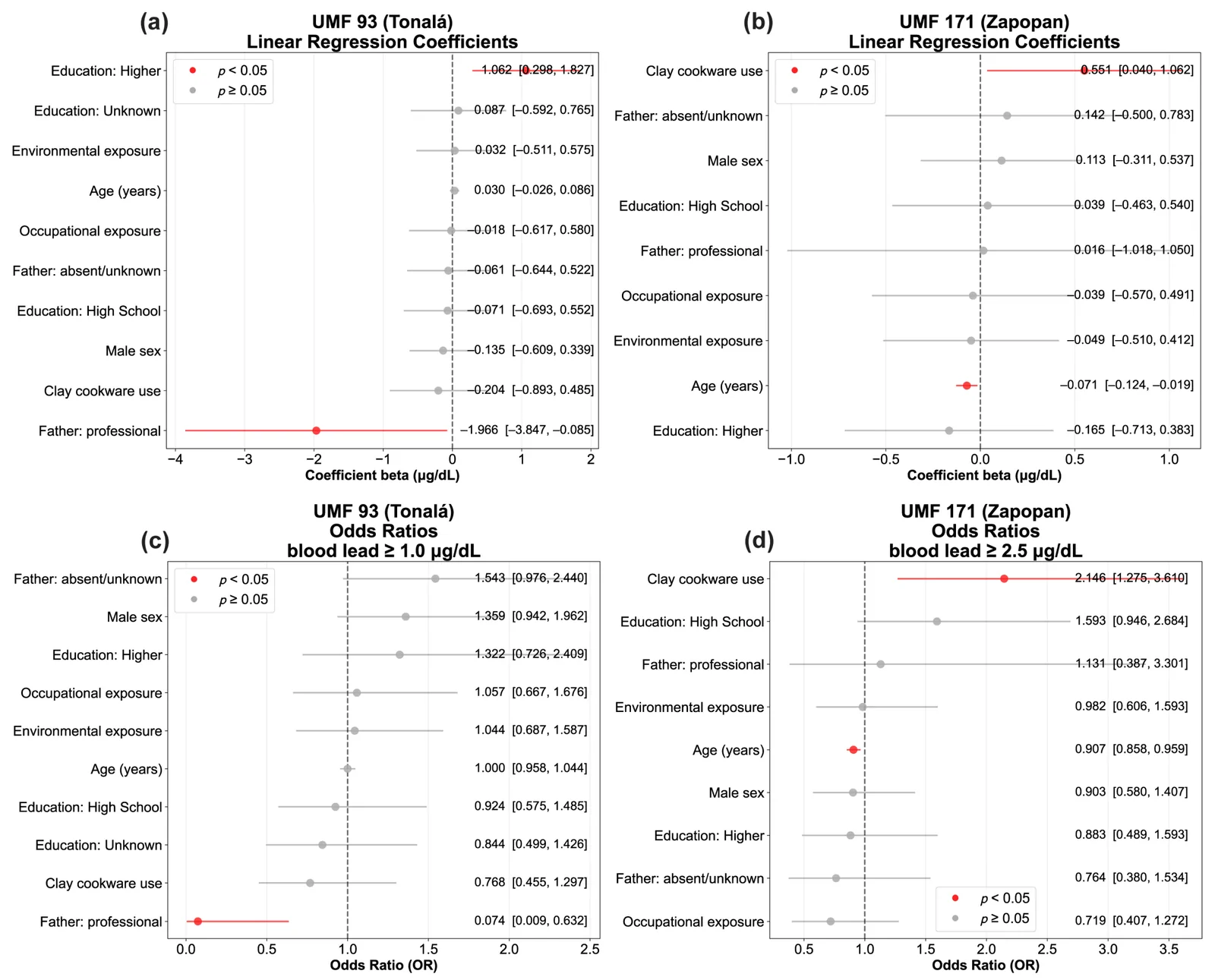
Multivariable models of blood lead level at the two study sites. (**a**,**b**) Adjusted coefficients (β) from multiple linear regression for UMF No. 93, Tonalá and UMF No. 171, Zapopan, respectively. (**c**,**d**) Adjusted odds ratios (OR) from logistic regression for the probability of exceeding a site-specific blood lead threshold, for the same two sites, respectively (≥1.0 µg/dL at UMF No. 93; ≥2.5 µg/dL at UMF No. 171). All models used the predictor specification described in Section 2.3. Points represent point estimates and horizontal lines represent 95% confidence intervals; red points indicate *p* < 0.05 and gray points indicate *p* ≥ 0.05. Dashed vertical lines mark the null value (β = 0 in **a**,**b**; OR = 1 in **c**,**d**).

In the linear models on the untransformed scale (Figure 9a,b), two predictors reached significance at UMF No. 93. Paternal professional occupation was associated with a 1.966 µg/dL lower blood lead level (95% CI −3.847 to −0.085), consistent in direction with the bivariate comparison. Higher parental education was associated with a 1.062 µg/dL higher level (95% CI 0.298 to 1.827), an estimate not corroborated by the descriptive data; median blood lead level in the higher-education group (1.23 µg/dL) was comparable to the other education categories (1.08 to 1.27 µg/dL; Table 1), and the bivariate comparison was not significant (*p* = 0.3017; Figure 5a). At UMF No. 171, glazed earthenware use was associated with a 0.551 µg/dL higher blood lead level (95% CI 0.040 to 1.062) and each additional year of age with a 0.072 µg/dL lower level (95% CI −0.124 to −0.019), both consistent with their bivariate results. No other predictor reached significance at either site.

In the logistic models (Figure 9c,d), the three corroborated predictors again reached significance, while parental education did not. At UMF No. 93, paternal professional occupation was associated with lower odds of exceeding 1 µg/dL (OR = 0.074, 95% CI 0.009–0.632). At UMF No. 171, glazed earthenware use was associated with higher odds of exceeding 2.5 µg/dL (OR = 2.146, 95% CI 1.275 to 3.610) and each additional year of age with lower odds (OR = 0.907, 95% CI 0.858 to 0.959). At the conventional 5 µg/dL threshold, no predictor reached significance at either site (Supplementary Figure S1). At UMF No. 93, the professional occupation category contained no children above this threshold and could not be estimated.

**Figure 10:**
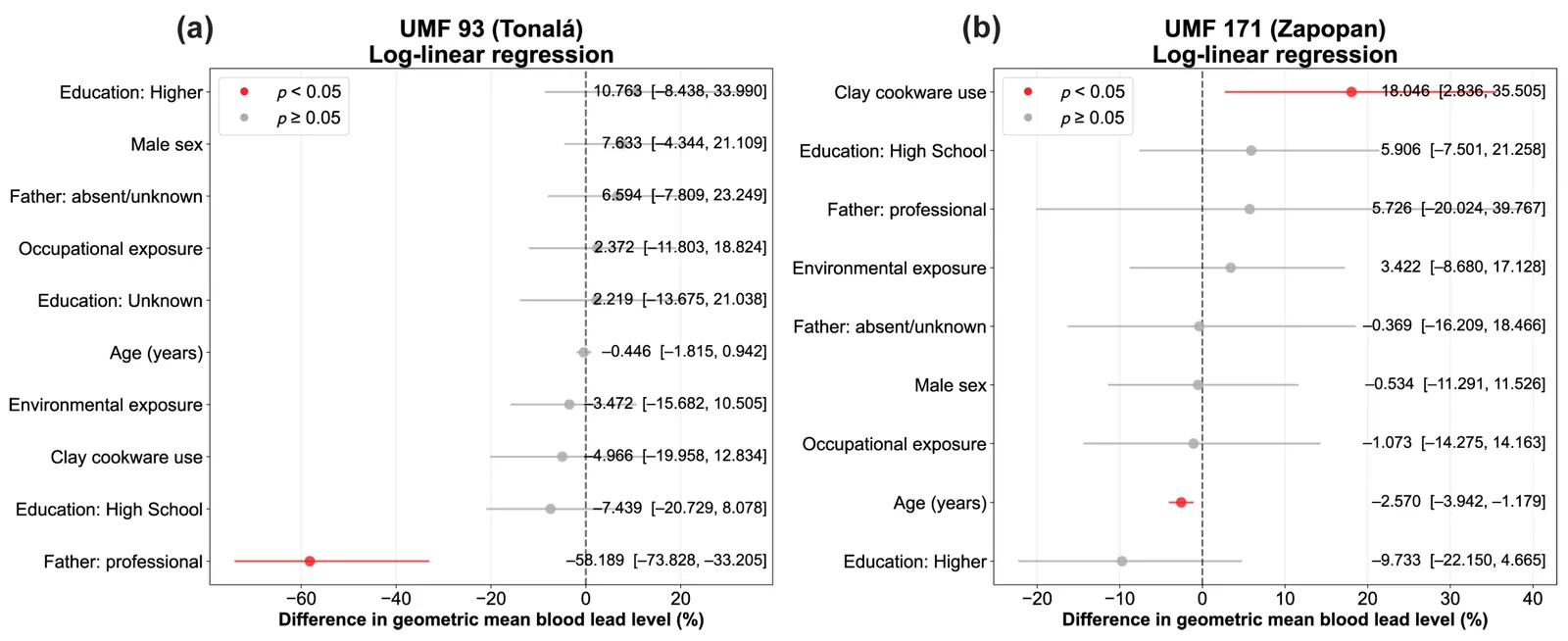
Adjusted coefficients from linear regression on the natural logarithm (Log-linear) of blood lead level, expressed as percent differences in geometric mean. (**a**) UMF No. 93, Tonalá. (**b**) UMF No. 171, Zapopan. Models used the same predictor specification as Figure 9. Points represent point estimates and horizontal lines 95% confidence intervals; red points indicate *p* < 0.05 and grey points *p* ≥ 0.05. The dashed vertical line marks the null value (0% difference).

Because the untransformed linear models are sensitive to the strong right skew of blood lead level at both sites, we also fitted them on the logarithmic scale, expressing effects as percent differences in geometric mean (Figure 10). At UMF No. 93, paternal professional occupation was associated with a 58.2% lower geometric mean blood lead level (95% CI −73.8 to −33.2), a larger and more precisely estimated effect than on the untransformed scale. At UMF No. 171, glazed earthenware use was associated with an 18.0% higher geometric mean (95% CI 2.8 to 35.5) and each additional year of age with a 2.6% lower geometric mean (95% CI −3.9 to −1.2). Parental education did not reach significance at either site. We assessed these models through residual, normality, homoscedasticity, and leverage diagnostics (Supplementary Figure S2). Residuals were approximately normally distributed with constant variance at both sites; no observation exceeded a Cook’s distance of 1, and observations with elevated leverage showed small residuals, indicating that no individual observation exerted disproportionate influence on the estimates.

## 4. Discussion

This study characterizes blood lead levels in children insured by the Mexican Social Security Institute (IMSS) at two Family Medicine Units in the metropolitan area of Guadalajara, Jalisco. The two municipalities differ in their exposure history: Tonalá is a traditional glazed-pottery center historically documented as a site of high lead exposure among the children of potters [9], while Zapopan is an urban municipality with a scarce pottery-making tradition where domestic use of earthenware nonetheless remains common, as in much of the country [6,17]. Applying the same protocol, instrument, and analytical specification at both sites allows their exposure profiles and associated factors to be compared directly within a population that shares the same tier of institutional healthcare.

Prevalence of BLL ≥5 µg/dL was low at both sites, 3.2% in Tonalá and 6.4% in Zapopan, well below the 17.4% national estimate for Mexican children aged 1–4 derived from the ENSANUT 100k extrapolation [7] and the 21.8% reported in localities under 100,000 residents [6]. This comparison should be read with caution, since the national figures apply to children aged 1–4, while our cohorts span 0–15 years and are weighted toward older children, in whom blood lead levels were lower at UMF No. 171. Measured against the current CDC reference value of 3.5 µg/dL rather than the older 5 µg/dL benchmark, the proportion affected doubles at both sites, to 6.6% in Tonalá and 16.3% in Zapopan. More striking still is how many children exceeded 1 µg/dL: 59.4% in Tonalá and 93.6% in Zapopan, with medians of 1.16 and 2.08 µg/dL. An early meta-analysis found no threshold in the relationship between blood lead and full-scale IQ, with effects persisting down to approximately this concentration and steeper slopes in studies of populations with lower mean levels [18]. A prospective birth cohort reported lower cognitive scores in boys with increasing cord blood lead at a geometric mean below 1 µg/dL [19], and the American Academy of Pediatrics has stated that no safe threshold has been identified [3]. Against this evidence, Mexico’s current regulatory limit of 10 µg/dL under NOM-199-SSA1-2000 [5], substantially understates the relevance of exposure in this population.

The prevalence observed in Tonalá is striking when set against its own history. A 1983 study reported a mean BLL of 39.5 µg/dL among the children of local potters, with 41–43% of participants above 40 µg/dL [9], figures far higher than those observed in our sample four decades later. This contrast suggests a substantial shift in the exposure pattern, plausibly attributable to the progressive rollout of national glazed-pottery substitution programs [20,21], the mandatory limits on lead release from pottery under Mexican normative NOM-231-SSA1-2016 [22], and the composition of the IMSS-insured population itself. Our findings also contrast with those from other Mexican states with more intense exposure: 14.7% of newborns in Morelos, in south-central Mexico, had BLL ≥ 5 µg/dL, rising to 22.2% in the state’s most marginalized municipalities [23], and pottery-making communities in Michoacán, a western state with its own ceramics tradition, showed mean BLL around 20–25 µg/dL among potters’ children, compared with 5–7 µg/dL among children from non-pottery-making communities [24].

Household glazed earthenware use illustrates this contrast directly. In Zapopan, it was independently associated with higher blood lead level across all three models, corresponding to an increase of 0.551 µg/dL on the absolute scale and an 18.0% higher geometric mean. Although modest in absolute terms, this effect represents a meaningful shift within a population whose geometric mean already sits at 2.14 µg/dL, pushing affected children further into a range the toxicological literature considers biologically active. This finding aligns with reports identifying glazed earthenware as the principal source of chronic pediatric lead exposure in Mexico [7,8,15], and with a recent study from Guanajuato, another Mexican state with a ceramics tradition, which found a fivefold increase in the odds of cord blood lead ≥3.5 µg/dL among children of mothers who reported using glazed ceramics [25]. In Tonalá, no such association emerged. Two explanations may account for this null result without indicating an absence of real effect. First, the binary use indicator cannot capture frequency or intensity of use, a dimension shown elsewhere to matter for BLL [7]. Second, parents in Tonalá may already have received guidance against using earthenware with acidic or hot foods, or locally produced pottery may already comply with the lead-release limits set by NOM-231-SSA1-2016.

In Zapopan, younger age was associated with higher BLL, both in the correlation analysis, across age groups, and in all three multivariable models, with each additional year corresponding to a 2.6% lower geometric mean. This pattern is consistent with greater intestinal lead absorption in early childhood and with behaviors characteristic of that developmental stage, such as hand-to-mouth activity and pica [2,26]. No age gradient was apparent in Tonalá, where blood lead level neither correlated with age nor differed across age groups. The two sites diverge on both age and glazed earthenware use, which may reflect a single pattern: an age gradient is expected where an active household exposure route is present, and glazed earthenware was associated with blood lead level only in Zapopan.

In Tonalá, paternal professional occupation was associated with lower blood lead level in all three models, with children of professionally employed fathers showing a 58.2% lower geometric mean than those in other occupational categories. This finding may reflect an underlying socioeconomic gradient, in which professional occupations are associated with housing, dietary, and household conditions that reduce the exposure routes characteristic of the municipality. Given that only nine fathers fell into this category, however, the estimate should be treated as exploratory, and the width of its confidence intervals reflects that limitation.

Parental education was also associated with blood lead level in Tonalá but on the absolute scale alone. This estimate was not supported by the descriptive data, the bivariate comparison, or the log-transformed model, and it illustrates the sensitivity of least-squares estimation to extreme values in a right-skewed distribution: the highest concentration in the cohort falls within the higher-education category. The log transformation, applied in response to that skew, removed the association.

Neither environmental nor occupational exposure indicators showed a significant association with BLL at either site, in either bivariate or multivariable analyses. Two explanations are complementary. First, both indicators are parent-reported and were not verified: environmental exposure rests on a self-identified source within 500 m of the home rather than on measured lead concentrations in soil, household dust, water, or air, parameters that Mexican studies using direct environmental sampling have linked to children’s BLL [27]. Second, occupational exposure was reported at a similar frequency at both sites, yet the occupations most closely tied to lead exposure in these communities, traditional pottery-making and informal recycling, fall largely outside formal employment and are therefore underrepresented in an IMSS-insured population. What the indicator captures is likely broader and less specific than the exposure pathways of concern.

This underrepresentation follows from where the IMSS-insured population sits within Mexico’s stratified health system. Social security institutions, IMSS among them, cover formal employees and their families through contributory affiliation, providing pensions, work-risk and disability insurance, and childcare alongside healthcare, and operate their own network of clinics and hospitals [28]. People outside formal employment receive healthcare through a separate publicly funded system with its own facilities but remain outside social security and its associated benefits. That second system has been restructured repeatedly, from Seguro Popular until 2019, to the Instituto de Salud para el Bienestar (INSABI) from 2020, and to IMSS-Bienestar from 2023, the last of which, despite its name, is administered separately from social security. Over this period the uninsured share rose nationally from 6.2% in 2016 to 29.1% in 2023, and in Jalisco from 6.7% in 2015 to 23.3% in 2023 [29], while entitlement to social security itself lapses with employment [30]. Lack of access to social security is itself one of the nine indicators by which poverty is officially measured in Mexico, alongside educational lag, housing quality, and access to food [31]; formal sector employment also carries higher and more stable income than the informal work on which most uninsured households depend [32]. Our findings therefore describe children whose families hold formal employment and cannot be generalized to the pediatric population of either municipality as a whole. That same restriction, however, is what makes the comparison informative: it holds social position approximately constant across the two sites, so the contrast between them reflects local exposure conditions rather than differences in socioeconomic composition.

Beyond the composition of the study population, several limitations should be considered when interpreting these findings. The exposure questionnaire was not formally validated, and all exposure indicators are parent-reported and dichotomous, capturing neither frequency nor intensity of contact. No environmental sampling was conducted, so the environmental indicator reflects reported proximity rather than measured exposure. The cross-sectional design precludes causal inference, and the associations identified here should be regarded as hypotheses for confirmation in longitudinal studies. Recruitment among children already scheduled for a complete blood count means participants were drawn from those attending clinical services rather than from the general insured population of either municipality. Finally, the two cohorts were recruited in different periods, so between-site comparisons should be interpreted as descriptive rather than as a controlled contrast.

## 5. Conclusions

Few children reached blood lead levels of 5 µg/dL (3.2% in Tonalá, 6.4% in Zapopan), roughly twice as many exceeded the current CDC reference value of 3.5 µg/dL (6.6% and 16.3%), and most exceeded 1 µg/dL (59.4% and 93.6%). Household glazed earthenware use emerged as a modifiable exposure source in Zapopan, associated with higher blood lead level across all three models, while in Tonalá the only factor consistently associated with lower levels was paternal professional occupation, an exploratory finding given its small subgroup. These results support a staged approach. In the short term, facility-level surveillance can be extended to units serving communities with known exposure sources, which this study shows to be feasible within routine care by adding lead determination to blood draws already scheduled. Data from multiple such units would build the distributional evidence base that criterion revision currently lacks in Mexico and would make visible the exposure that the present threshold of 10 µg/dL does not capture; fewer than 1% of children in our cohorts reached that threshold, while most were within the range in which cognitive effects have been documented. Because our design draws on a formal-sector population and relies on parent-reported exposure indicators, confirmation will require longitudinal studies with repeated blood lead measurement, recruitment extending beyond social security coverage, and direct environmental sampling of soil, household dust, and water.

## Figures and Tables

**Table 1:** Characteristics of the study populations and blood lead levels at UMF No. 93 (Tonalá) and UMF No. 171 (Zapopan), with between-site and within-site comparisons.

Variable	UMF 93 (Tonalá) *n* (% [95% CI]	UMF 93 (Tonalá) BLL median [IQR]	UMF 171 (Zapopan) *n* (% [95% CI]	UMF 171 (Zapopan) BLL Median [IQR]	Between-Site *p*	Within-Site BLL Tonalá *p*	Within-Site BLL Zapopan *p*
*n*	500	—	374	—	—	—	—
Sex	—	—	—	—	*p* = 0.0349	*p* = 0.0722	*p* = 0.9828
Female	266 (53.2% [48.8–57.5])	1.08 [0.74–1.75]	172 (46.0% [41.0–51.1])	2.06 [1.47–3.05]	—	—	—
Male	234 (46.8% [42.5–51.2])	1.23 [0.88–1.78]	202 (54.0% [48.9–59.0])	2.09 [1.48–2.92]	—	—	—
Age group	Median 10 [6–13] y	—	Median 8 [5–11] y	—	*p* = 0.0005	*p* = 0.7032	*p* = 0.0043
Infancy (<1)	20 (4.0% [2.6–6.1])	1.30 [0.88–1.96]	15 (4.0% [2.4–6.5])	2.55 [1.87–4.19] ^ab	—	—	—
Toddler (1–2)	30 (6.0% [4.2–8.4])	1.06 [0.79–1.90]	28 (7.5% [5.2–10.6])	2.46 [1.59–3.43] ^ab	—	—	—
Early childhood (3–5)	68 (13.6% [10.9–16.9])	1.26 [0.86–1.71]	60 (16.0% [12.7–20.1])	2.61 [1.88–3.21] ^a	—	—	—
Middle childhood (6–11)	209 (41.8% [37.6–46.2])	1.16 [0.78–1.67]	180 (48.1% [43.1–53.2])	1.98 [1.47–2.68] ^b	—	—	—
Adolescence (12–15)	173 (34.6% [30.6–38.9])	1.12 [0.73–1.77]	91 (24.3% [20.3–28.9])	1.90 [1.35–2.80] ^b			
BLL risk category (µg/dL)	—	—	—	—	—	—	—
<1	203 (40.6% [36.4–45.0])	—	24 (6.4% [4.3–9.4])	—	—	—	—
1–3.5	264 (52.8% [48.4–57.1])	—	289 (77.3% [72.8–81.2])	—	—	—	—
3.5–5	17 (3.4% [2.1–5.4])	—	37 (9.9% [7.3–13.3])	—	—	—	—
5–10	13 (2.6% [1.5–4.4])	—	21 (5.6% [3.7–8.4])	—	—	—	—
≥10	3 (0.6% [0.2–1.7])	—	3 (0.8% [0.3–2.3])	—	—	—	—
BLL summary (µg/dL)	—	—	—	—	*p* = <0.0001	—	—
Median [IQR]	—	1.16 [0.80–1.77]	—	2.08 [1.48–3.00]	—	—	—
Geometric mean	—	1.22	—	2.14	—	—	—
Min	—	0.22	—	0.24	—	—	—
Max	—	53.51	—	22.75	—	—	—
Prevalence at threshold	—	—	—	—	—	—	—
≥1 µg/dL	297 (59.4% [55.0–63.6])	—	350 (93.6% [90.6–95.7])	—	*p* = <0.0001	—	—
≥3.5 µg/dL	33 (6.6% [4.7–9.1])	—	61 (16.3% [12.9–20.4])	—	*p* = <0.0001	—	—
≥5 µg/dL	16 (3.2% [2.0–5.1])	—	24 (6.4% [4.3–9.4])	—	*p* = 0.0243	—	—
≥10 µg/dL	3 (0.6% [0.2–1.7])	—	3 (0.8% [0.3–2.3])	—	*p* = 0.7051	—	—
Environmental exposure	—	—	—	—	*p* = <0.0001	*p* = 0.9739	*p* = 0.4330
Not exposed	136 (27.2% [23.5–31.3])	1.16 [0.76–1.96]	259 (69.3% [64.4–73.7])	2.08 [1.46–2.97]	—	—	—
Exposed	364 (72.8% [68.7–76.5])	1.15 [0.82–1.69]	115 (30.7% [26.3–35.6])	2.10 [1.60–3.01]	—	—	—
Occupational exposure	—	—	—	—	*p* = 0.8879	*p* = 0.8267	*p* = 0.5396
Not exposed	399 (79.8% [76.1–83.1])	1.15 [0.78–1.81]	297 (79.4% [75.0–83.2])	2.11 [1.50–3.04]	—	—	—
Exposed	101 (20.2% [16.9–23.9])	1.17 [0.84–1.66]	77 (20.6% [16.8–25.0])	2.06 [1.47–2.81]	—	—	—
Glazed earthenware use	—	—	—	—	*p* = 0.0281	*p* = 0.2520	*p* = 0.0244
Does not use	416 (83.2% [79.7–86.2])	1.17 [0.81–1.76]	289 (77.3% [72.8–81.2])	2.02 [1.47–2.85]	—	—	—
Uses	84 (16.8% [13.8–20.3])	1.06 [0.70–1.87]	85 (22.7% [18.8–27.2])	2.39 [1.79–3.21]	—	—	—
Cumulative exposure burden	—	—	—	—	—	*p* = 0.5232	*p* = 0.4002
None	72 (14.4% [11.6–17.7])	1.13 [0.80–1.92]	169 (45.2% [40.2–50.3])	1.96 [1.42–2.85]	*p* = <0.0001	—	—
One	316 (63.2% [58.9–67.3])	1.19 [0.79–1.79]	137 (36.6% [31.9–41.6])	2.10 [1.56–3.06]	*p* = <0.0001	—	—
Two or more	112 (22.4% [19.0–26.3])	1.08 [0.82–1.63]	68 (18.2% [14.6–22.4])	2.14 [1.62–2.85]	*p* = 0.1271	—	—
Parental education	—	—	—	—	—	*p* = 0.3017	*p* = 0.0691
Basic	169 (33.8% [29.8–38.1])	1.15 [0.82–1.81]	123 (32.9% [28.3–37.8])	2.06 [1.52–2.75]	*p* = 0.7772	—	—
High School	130 (26.0% [22.3–30.0])	1.08 [0.77–1.53]	143 (38.2% [33.5–43.3])	2.35 [1.60–3.11]	*p* = 0.0001	—	—
Higher Education	77 (15.4% [12.5–18.8])	1.23 [0.74–1.67]	108 (28.9% [24.5–33.7])	1.96 [1.37–2.94]	*p* = <0.0001	—	—
Unknown	124 (24.8% [21.2–28.8])	1.27 [0.85–1.97]	0 (0.0% [0.0–1.0])	—	—	—	—
Paternal occupation	—	—	—	—	—	*p* = 0.0011	*p* = 0.9337
Employee	255 (51.0% [46.6–55.4])	1.15 [0.80–1.81] ^a	210 (56.1% [51.1–61.1])	2.03 [1.47–2.93]	*p* = 0.1311	—	—
Commerce/Industry	82 (16.4% [13.4–19.9])	1.00 [0.71–1.41] ^ab	100 (26.7% [22.5–31.4])	2.13 [1.47–3.03]	*p* = 0.0002	—	—
Professional	9 (1.8% [0.9–3.4])	0.74 [0.30–0.80] ^b	17 (4.5% [2.9–7.2])	2.13 [1.63–3.06]	*p* = 0.0181	—	—
Unknown/Absent	154 (30.8% [26.9–35.0])	1.30 [0.86–1.86] ^a	47 (12.6% [9.6–16.3])	2.07 [1.56–2.77]	—	—	—
Maternal occupation	—	—	—	—	—	*p* = 0.7697	*p* = 0.5056
Homemaker	111 (22.2% [18.8–26.0])	1.16 [0.82–1.66]	137 (36.6% [31.9–41.6])	2.14 [1.54–2.92]	*p* = <0.0001	—	—
Employee	248 (49.6% [45.2–54.0])	1.16 [0.77–1.77]	152 (40.6% [35.8–45.7])	2.03 [1.48–3.04]	*p* = 0.0085	—	—
Commerce/Industry	34 (6.8% [4.9–9.4])	1.25 [0.89–2.04]	56 (15.0% [11.7–18.9])	2.21 [1.57–3.00]	*p* = <0.0001	—	—
Professional	10 (2.0% [1.1–3.6])	1.25 [0.92–1.63]	28 (7.5% [5.2–10.6])	1.96 [1.47–2.79]	*p* = <0.0001	—	—
Unknown/Absent	97 (19.4% [16.2–23.1])	1.06 [0.79–1.80]	1 (0.3% [0.0–1.5])	0.56 [0.56–0.56]	—	—	—

## Data Availability

The data presented in this study are available from the corresponding authors upon reasonable request.

## Supplementary Materials

The following supporting information can be downloaded at: https://www.mdpi.com/article/10.3390/toxics14090744/s1, Figure S1: Adjusted odds ratios from logistic regression for exceeding 5 µg/dL; Figure S2: Diagnostic plots for the log-linear regression models at the two study sites.

## Abbreviations

The following abbreviations are used in this manuscript:

BLL	Blood lead level
CDC	Centers for Disease Control and Prevention
CI	Confidence interval
EDTA	Ethylenediaminetetraacetic acid
ENSANUT	Encuesta Nacional de Salud y Nutrición (National Health and Nutrition Survey)
GM	Geometric mean
IMSS	Instituto Mexicano del Seguro Social (Mexican Social Security Institute)
INSABI	Instituto de Salud para el Bienestar (Health Institute for Welfare)
IQR	Interquartile range
LOD	Limit of detection
LOQ	Limit of quantification
Md	Median
NOM	Norma Oficial Mexicana (Official Mexican Standard)
ns	Not significant
OR	Odds ratio
SD	Standard deviation
SECIHTI	Secretaría de Ciencia, Humanidades, Tecnología e Innovación
StaR	Standards for Research in Child Health
UMF	Unidad de Medicina Familiar (Family Medicine Unit)
