# Supplementary material for: Blood Lead Levels and Associated Sociodemographic and Occupational Factors in Children from Two Mexican Communities: A Comparative Study
Source: Toxics. 2026 Aug 23;14(9):744. doi: 10.3390/toxics14090744 (PMC13611243; doi:10.3390/toxics14090744)
Supplement: Supplementary file 1 [file toxics-14-00744-s001.zip › toxics-4459570-supplementary.pdf]

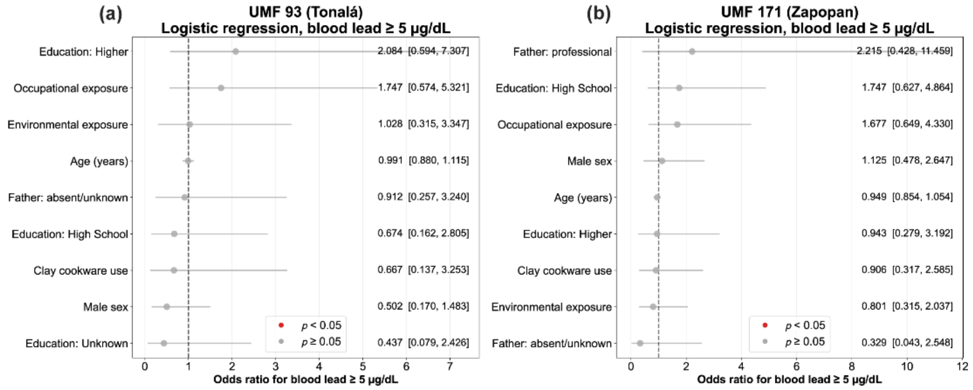

Figure S1. Adjusted odds ratios from logistic regression for exceeding 5 μg/dL. (a) UMF No. 93, Tonalá. (b) UMF No. 171, Zapopan. Models used the same predictor specification as Figure 9. Points represent point estimates and horizontal lines 95% confidence intervals. The dashed vertical line marks OR=1. The professional occupation category at UMF No. 93 contained no children above the threshold and is not shown.

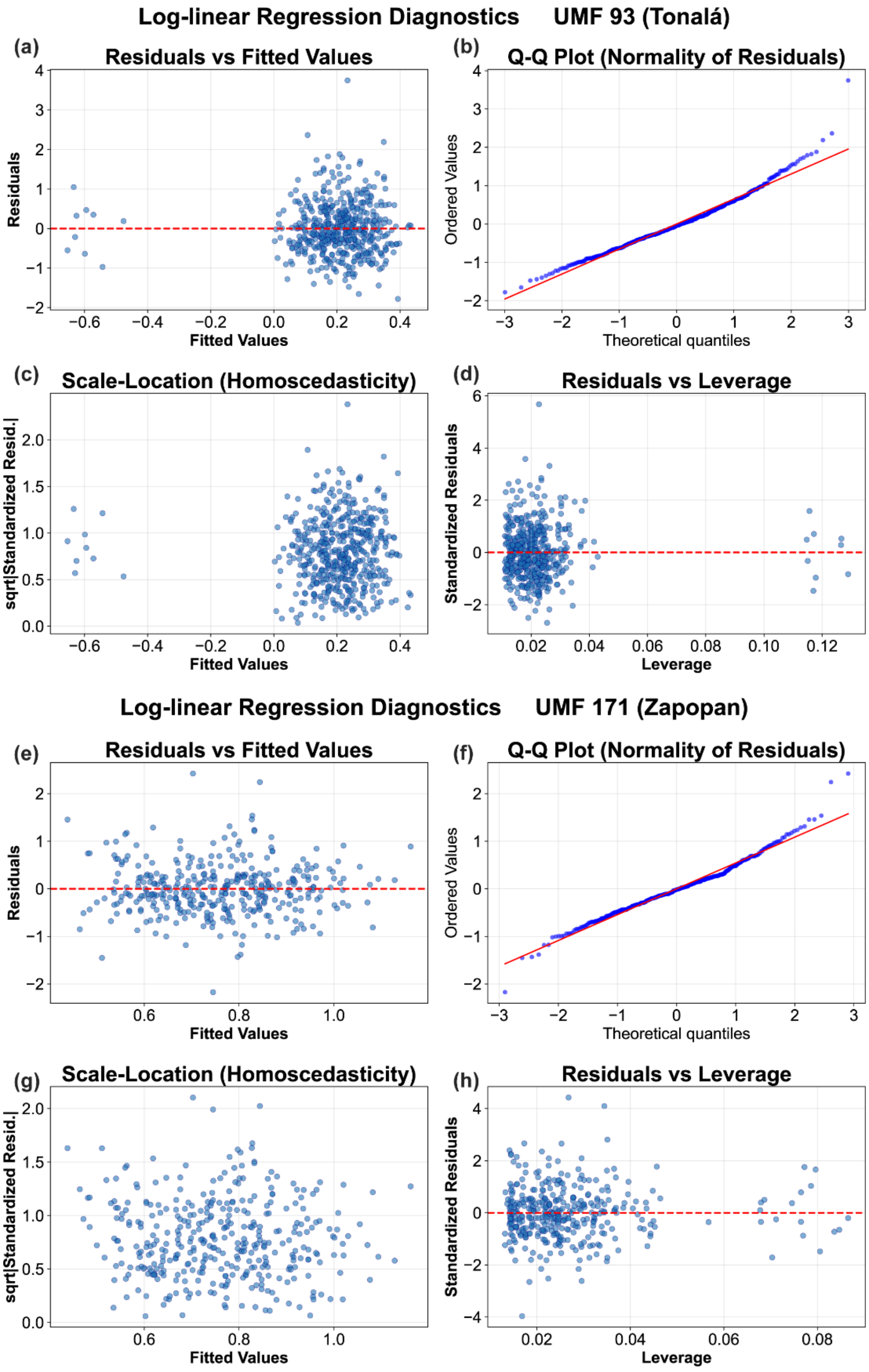

Figure S2. Diagnostic plots for the log-linear regression models at the two study sites. (a-d) UMF No. 93, Tonalá. (e-h) UMF No. 171, Zapopan. For each site, four panels are shown. Residuals against fitted values (a, e) assess linearity and the absence of systematic structure; residuals should scatter randomly about zero, indicated by the

dashed red line. Normal quantile-quantile plots (b, f) assess the normality of residuals; points falling along the reference line indicate approximate normality. Scale-location plots (c, g) assess homoscedasticity by plotting the square root of the absolute standardized residuals against fitted values; a uniform spread indicates constant variance. Residuals against leverage (d, h) identify observations with unusual predictor values that may exert disproportionate influence on the fitted coefficients. At UMF No. 93, the isolated cluster of low fitted values in panels (a) and (c), and the corresponding high-leverage points in panel (d), correspond to the professional paternal occupation category (n=9).
